# Sexist Attitudes in Adolescents: Prevalence and Associated Factors

**DOI:** 10.3390/ijerph191912329

**Published:** 2022-09-28

**Authors:** Elena Vila-Cortavitarte, N. Marta Díaz-Gómez, José Miguel Díaz-Gómez

**Affiliations:** 1 Programa de Doctorado en Ciencias de la Salud, Línea Materno-Infantil, Universidad de la Laguna (Tenerife), 38200 San Cristóbal de La Laguna, Spain; 2 Facultad de Ciencias de la Salud, Universidad de la Laguna (Tenerife), 38200 San Cristóbal de La Laguna, Spain; 3 Facultad de Psicología, Universidad de la Laguna (Tenerife), 38200 San Cristóbal de La Laguna, Spain

**Keywords:** sexism, gender identity, pornography, adolescent, gender-based violence, gender equity

## Abstract

Gender violence is a major public health issue. The aim of this study was to determine the prevalence of sexist attitudes that could be associated with said violence, and to identify some sociodemographic variables that predict sexism. A cross-sectional observational study was conducted with 723 adolescents between the ages of 14 to 19. Their explicit sexist attitudes were measured with the EVAMVE and EARG scales, and their implicit attitudes were measured with a series of assessment items regarding the behavior of the protagonists of a video and a story in which a young couple interacts. Explicit and implicit sexist attitudes were detected in adolescents of both sexes. Qualitatively, the assessment of the behavior of the female protagonist is striking. Regarding the sexism predictors, it was found that male adolescents, those born outside of Spain, those who were studying in a public school, those whose parents did not have university studies, and those who consumed the most pornography presented attitudes that were significantly more sexist. These results suggest that it is necessary to strengthen education in equality and prevention of gender violence in adolescents, and to develop affective-sexual education programs.

## 1. Introduction

Gender-based violence is a major social and public health problem. It is present in all population groups, including adolescents, and is related to the existence of a patriarchal society structured from gender inequality [1,2,3,4]. A report by the European Agency for Fundamental Rights (FRA) in 2014 reveals that the percentage of women over 15 years of age who claimed to have been victims of physical or sexual violence was 33% in Europe and 22% in Spain [5]. A systematic review from 2017 on the prevalence of gender violence during dating shows that in adolescence there are very high percentages of psychological aggression and that the rates of aggressive behavior in the case of adolescents are slightly higher than those of young adults [6].

In 2018, the Help Foundation for children and adolescents at risk (ANAR) published the trend in violence suffered by children and adolescents between 2009 and 2016, based on the calls received by helplines. The data reflects a very high growth rate, from 210 cases of gender violence dealt with in 2009 to 1643 in 2016. This study also shows that new technologies are the main means of exercising violence [7].

New technologies facilitate access to online pornography, which is related to a greater propensity to have sexist attitudes, and to perpetrate sexual coercion and abuse [8]. The most worrying aspect of access to pornographic content is that it teaches young males sexist attitudes and behaviors, such as belittling women, sexualizing female pain, not questioning females’ desire, or turning sex into an obligation associated with the domination over women. Alternatively, young females learn that their pleasure is secondary, and that they must show an unconditional (and sometimes submissive) willingness to engage in sexual intercourse. In addition, pornography enhances unacceptable situations of inequality, such as sexual violence [9].

According to data from the National Institute of Statistics (INE), the number of victims of gender violence in Spain under 18 years of age increased between 2020 and 2021 by 28.6%, and this population group showed the most significant increase [10]. All this shows that gender violence in adolescence is increasing, with serious repercussions for the health of young women [1,10]. When a female adolescent finds herself in a relationship of control and domination, the risks increase of consuming toxic substances, poor school performance, difficulties in relationships with peers, and other behavioral problems, in her affective and sexual life [1].

Gender violence has a multicausal origin, associated with a combination of sociocultural and individual factors. Physical force is not the only means of exerting gender violence. On the contrary, sexism is one of its most deeply rooted forms of expression. The Committee of Ministers of the Council of Europe, in the recommendation to prevent and combat sexism, defines sexism as any act, gesture, visual representation, oral or written statement, practice or behavior, based on the idea that a person or group of people is lower because of their sex [11]. This approach to sexism can be identified as the classic conception. From the 1990s onwards, other, more articulated theoretical proposals have emerged, parallel to the advance in social movements for equality. These proposals include that of Swim et al. [12], based on the distinction between an archaic sexism and a new sexism. The latter includes, along with the denial of discrimination against women, a certain latent discomfort at the loss of power of men. In the same sense is the proposal of Tougas et al. [13], based on the concept of neo-sexism, which is also modulated by the conflict between the unstoppable social process of equalization between the sexes, and mistrust of its long-term consequences. Subsequently Glick and Fiske [14,15] proposed the idea that sexism has a two-dimensional character, with a hostile and a benevolent component. The hostile sexism suffered by women presents them as inferior. The benevolent side of sexism against women places the emphasis on aspects such as their alleged need for protection, while the sexism they suffer precisely highlights their ability to provide such protection. This two-dimensional conceptualization of sexism helps to understand its high prevalence in otherwise relatively advanced societies such as the case of Europe [16]. Leaper and Brown [17] concluded that, despite the efforts over the last fifty years to reduce gender inequality, sexism is still prevalent among adolescents.

There are other concepts close to sexism. The sexist attitude, according to Díaz Aguado [18], is the predisposition to respond with a certain kind of affective, cognitive, or behavior response depending on the sex of a subject. In a study framed within the Lights4violence project, Ayala et al. [19] detected sexist attitudes in adolescents of both sexes, with males reporting higher levels of hostile and benevolent sexism. Sexist beliefs, on the other hand, are the ideas or thoughts about attributes or characteristics assigned to subjects based on their sex. Very often these types of beliefs undervalue women and justify discrimination and violence.

Despite the relevance of the problem, few previous studies have analyzed the factors related to sexist attitudes in adolescents, which are of great importance in terms of prevention. This was the motivation for conducting this study. In this regard, the objective was to determine the prevalence of sexist attitudes in this age group, and to identify some predictive sociodemographic variables of sexism.

## 2. Materials and Methods

### 2.1. Study Design

A cross-sectional observational study, was carried out from 1 October 2019 to 8 January 2020.

### 2.2. Population and Sample

The participants were recruited through quota sampling carried out in high schools in the province of Tenerife, where 75% of the schools are public and 25% are private. In order to maintain this proportion in the sample, 3 public schools and 1 private school were selected. The inclusion criteria were: adolescent between 14 and 19 years old, studying at high school in one of the 4 schools selected for the study, and who agreed to participate. Subjects with a disability or language barrier that prevented them from understanding and completing the questionnaires were excluded.

The estimation of the sample size was carried out with the sample calculator in Excel [20], taking into account that in the 2017/2018 academic year, 14,088 adolescents attended high school in Tenerife [21]. Assuming a confidence level of 99%, a beta error of 5%, an expected proportion of 50%, and an expected loss proportion of 12%, the result was a sample of 720 adolescents (723 were used). Their mean age was 16.29 ± 0.7 years old. None of the subjects studied by the participants during the previous year included any content about sexual education or gender equality. The descriptive data of the sample can be seen in Table 1.

### 2.3. Procedure

Data collection was carried out by the main researcher, in person, in the classrooms of the selected high schools, during school hours and after reading the instructions. The subjects had 30 min to respond to a self-completed questionnaire that included questions on the following aspects: age, sex, country of birth, high school year, school, type of high school, level of education, marital status and employment status of parents, and consumption of pornography.

Two scales were included in the questionnaire: one to measure explicit sexist attitudes, violence and gender stereotypes in adolescents (EVAMVE), designed and validated in February 2018 by Marchal Torralbo et al. [22], with a Cronbach’s alpha coefficient of 0.878; and another to assess explicit gender role attitudes (EARG), designed and validated in July 2014 by García Cueto et al. [23], with a Cronbach’s alpha coefficient of 0.99. Both scales consist of 20 items. Each item was evaluated on a 5-point Likert-type scale, ranging from total disagreement (1) to total agreement (5), with a neutral central value. The final score of the scale was obtained by adding the values of each item, so that the higher the score, the greater the sexist attitudes.

The EVAMVE and EARG scales measure the verbal or explicit component of sexist attitudes. Since these scales may be somewhat influenced by social desirability, we decided to also include an assessment of implicit sexist attitudes, following the recommendation to complement both types of records in attitudinal studies [24,25,26]. For this purpose, in the same session, students were asked to answer 4 questions, to express their opinion about the video “I am ordinary” by Cloé F. Thompson (available at: https://vimeo.com/205412604, accessed on 13 June 2019) [27] and another 4 questions on a short, self-made story (Appendix A). Both the video and the short story show an interaction between two young people, a boy who forces an unwanted sexual relationship, and a girl who adopts a passive attitude. Participants were asked to give their opinions on the extent to which these two episodes (the video and the story) reflected gender-based violence or sexual abuse, and to evaluate the behavior of their protagonists. The questions were of this nature: “Please rate the young man’s behavior on a scale of 1 to 10, where 1 indicates that you found the behavior extremely inappropriate and 10 indicates that it is completely correct behavior”. We assumed that higher scores were representative of greater (implicit) sexist attitudes. The presentation of the different tests was balanced: half of the participants answered the scales first and then watched and evaluated the video and the story, while the other half did so in the reverse order.

The study was approved by the Ethics Committee of the University of La Laguna, and received the permission of the schools. In order to guarantee the rights of the students, their approval and their parents or legal guardians’ permission were requested, through informed consent. The treatment of the data was carried out in such a way as to guarantee confidentiality. The statistical analysis was performed with SPSS Statistics for Windows (Version 25.0. Armonk, NY, USA: IBM Corp.).

## 3. Results

The EVAMVE scale generated a Cronbach’s alpha equal to 0.86, with a mean score of 28.2 ± 7.5. The EARG scale generated a Cronbach’s alpha equal to 0.80, with a mean of 30.0 ± 8.1. The correlation between both scales was r_xy_ = 0.7 (*p* = 0.001). As previously mentioned, the evaluation of the implicit attitudes was carried out through eight items in which the participants gave their opinion about a video and a story that reflected gender violence. Due to the exploratory nature of this evaluation of implicit attitudes, we performed a factorial analysis with the eight indicators, since a priori, we did not know the extent to which the participants’ judgments were going to discriminate between the different aspects mentioned above (gender violence, sexual abuse, and correction of the behavior of both protagonists). Furthermore, this factorial analysis reduced the number of indicators, meaning the analysis was more easily interpreted. The result of the factorial analysis with Varimax rotation is summarized in Table 2. We obtained three relevant dimensions (with eigenvalues greater than 1), in which the items are grouped in an easily intelligible way. The first factor corresponds to the detection of gender violence and sexual abuse; the second to the evaluation of the behavior of the male protagonist; and the third to the evaluation of the behavior of the female protagonist. Regarding the latter, a high proportion of adolescents (81% in the video and 62% in the story) considered that the passive behavior of the female protagonist is incorrect, when she is forced to have an unwanted sexual relationship.

Next, we analyzed the impact of the sociodemographic variables on the two measures of explicit sexist attitudes (the EVAMVE and EARG scales) and the three measures of implicit attitudes (the three dimensions resulting from the factorial analysis). Table 3 shows the contrasts that became significant, based on t-tests for independent groups. The most powerful factor associated with levels of sexism, both implicit and explicit, is the gender of the participants. In our study, male adolescents obtained higher scores in the scales used in our study, compared to women, which indicates greater sexism. The country of origin also has significant effects on explicit attitudes: participants from Spanish families are less sexist than those whose families come from other countries. Among the latter, adolescents were mostly from Venezuela, Colombia, Italy, and Uruguay. Finally, it is worth mentioning certain effects of the sociodemographic indicators of an academic nature: the type of center (public or private) and the educational level of the parents (university/non-university). As can be seen in Table 3, there is less sexism among students at private schools and those students whose parents studied at university.

The other sociodemographic variables (age, years of stay in Spain, high school course, type of high school, maternal and paternal employment status, and maternal and paternal marital status) were not associated with significant differences, nor with the scales used to measure explicit attitudes or implicit attitudes. It should also be noted that the third dimension resulting from the factorial analysis (the evaluation of the behavior of the female protagonist) was not sensitive to any of the variables considered. Finally, the influence of another possible attitudinal predictor was analyzed, i.e., the level of exposure to pornographic content, evaluated through some items used by Sanjuán in his study on the consumption of pornography in Spanish adolescents [9], collected in Table 4.

Of the total participants, 84.8% responded affirmatively to the question of whether they had ever consumed pornography, 35.7% indicated that they had consumed pornography in the past 30 days, 11.1% considered that the sexual practices they had seen in pornography were similar to normal sexual practices, and 36.5% indicated that pornography influenced their sexual relations with their partner. We averaged the answers to these four items, computing “Yes” as 1, and “No” as 0, so that the final score in this variable, which we called “Exposure to pornography” is: 0, when the subject answers with a “no” to all the questions; 0.25 when they answer with a “yes” to one of them; 0.50 when they answer “yes” to two; 0.75 when they answer “yes” to three questions; and 1 when they answer positively to all (see Table 5).

**Figure 1:**
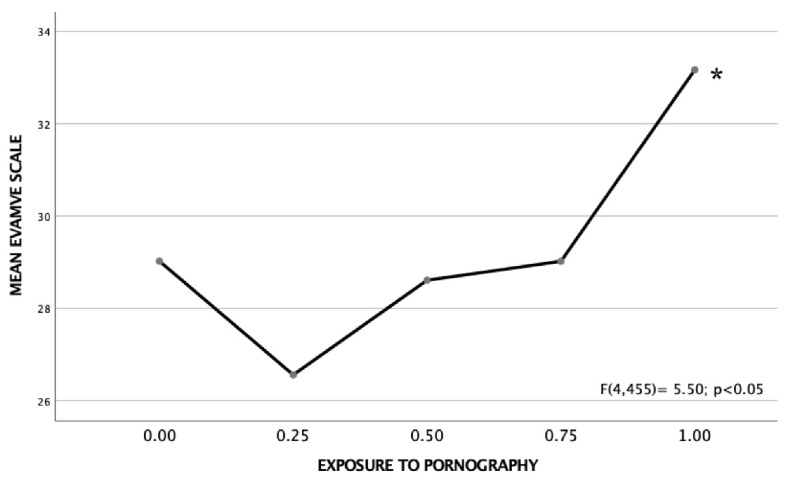
Mean of the EVAMVE scale according to the degree of exposure to pornography (minimum 0, maximum 1). The higher the score, the greater the explicit sexist attitudes, and violence and gender stereotypes. (* Marks the group that presents significant differences from the rest of the groups; Tukey’s HSD (*p* < 0.05).

**Figure 2:**
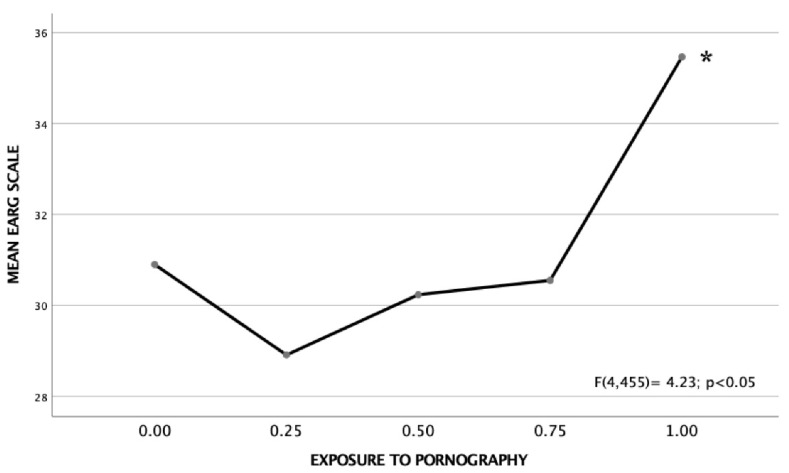
Mean of the EARG scale according to the degree of exposure to pornography (minimum 0, maximum 1). The higher the score, the greater the explicit gender role attitudes. (* Marks the group that presents significant differences from the rest of the groups; Tukey’s HSD (*p* < 0.05).

Using one-way analysis of variance, we found that exposure to pornography has a significant effect on explicit sexist attitudes. As can be seen in Figure 1 and Figure 2, the adolescents with the greatest exposure to pornographic content, that is, those who answered positively to the four items, presented a significantly higher score on both scales than the rest of the sample, as corroborated by Tukey’s HSD a posteriori analyses (*p* < 0.05).

## 4. Discussion

Our study was carried out with the purpose of determining the prevalence of sexist attitudes in adolescents and to identify some sociodemographic variables related to sexism. We found that a high percentage of adolescents have explicit and implicit sexist attitudes, have been exposed to pornographic content, and consider the passive behavior of females to be incorrect when they are forced to have an unwanted sexual relationship. Sexist attitudes were more accentuated in male adolescents, those born outside of Spain, those who had had greater exposure to pornographic content, those who studied in public educational schools, and those whose parents did not have university studies.

Numerous previous studies [2,3,6,22,23,24,28,29,30,31,32,33,34,35,36,37,38,39,40,41,42] conducted in adolescents and young adults of both sexes, using a wide variety of measurement instruments, are consistent with our findings in identifying explicit sexist attitudes, including threats, control tactics, prejudice, and discrimination against women. In our study, we also detected implicit sexist attitudes. In this sense, the judgement made by many students of the passive behavior of the female protagonist in the story and in the video, when forced to have an unwanted sexual relationship, is striking. Although our data do not allow us to identify the aspects of the girl’s behavior that generated the negative opinion of the participants, we believe that this result is in itself an indicator of a somewhat sexist assessment of the girl’s behavior.

Few authors have included instruments to measure implicit sexist attitudes [24], as we used in our study. The measurement of these implicit attitudes allows an evaluation of sexism that is less influenced by external control mechanisms and is more difficult to correct or adjust to social expectations. Cardenas M. et al. [24], in a study investigating the presence of explicit and implicit sexist attitudes, detected significant differences between men and women in implicit attitudes, but not in explicit attitudes. These results showed men had a more negative attitude towards women, which could indicate that the apparent decrease in sexism may cover up the fact that negative attitudes remain at an implicit level.

Most studies that analyze sexist attitudes using the EVAMVE scale, which was used in our study [22], or other evaluation instruments [31,32,33,34], are in accordance with our study in verifying more attitudes of this type in male adolescents and young men. By comparison, authors who analyzed the different types of sexism found significantly higher scores in male adolescents compared to females for traditional sexism [16,35], hostile sexism [36,37] and ambivalent sexism [16,38,39,40,41].

Our results show that adolescents with university-educated parents had a lower implicit sexist attitude. These findings are in agreement with those obtained by Ayala et al. [19], in a study of 12–17-year-old adolescents from different European countries, in which lower levels of benevolent sexism were detected in girls with university-educated mothers. Another study in Belgium also found that adolescents showed lower levels of benevolent and hostile sexist attitudes when their parents had a high level of education [43].

Coinciding with Arnoso et al. [42], we found significantly higher scores on the EVAMVE and EARG scales, which reflect more sexist explicit attitudes in adolescents born outside of Spain compared to those born in Spain; however, it is noteworthy that we found significant differences in the items that assess implicit sexist attitudes. Since explicit attitudes are more conditioned by social desirability than implicit attitudes, a possible explanation for this fact is that Spanish adolescents tend to answer what they consider to be socially correct in the questionnaires. Therefore, the question remains as to whether the higher scores on the EVAMVE and EARG scales, recorded among young people from other countries, reflect less “familiarity” with the prevailing social expectations in Spain, or a genuine difference in implicit sexist attitudes.

Furthermore, and similar to previous studies [8,9,44], we identified that there is a high percentage of adolescents who consume pornography (only 10.9% of the subjects presented a zero level of pornography consumption), and that they report that pornography influences their relationships. In our study, coinciding with other authors [8,44,45,46], we verified that the consumption of pornography is one of the variables that seems to enhance sexist attitudes. New studies are needed to determine if having sexist attitudes influences adolescents to consume more pornography, or if pornography causes them to have more sexist attitudes. Future studies could expand the age range to include subjects in early adolescence and record the gender identity of the respondents, in order to evaluate the influence of these variables, which is one of the limitations of our study. In the reviewed literature we found disparities in the measurement instruments, the measurement objectives (behavior, attitudes), and the timing of the measurements, which makes it difficult to offer a more precise comparison with the results of our study. As other authors point out [47], it is necessary to reach a consensus to use comparable measurement instruments and objectives.

Sexism is a problem that needs a comprehensive and systematic approach in adolescents. This is because sexist attitudes are related to harmful forms of intimate interaction between adolescents, with attitudes that tend to partner violence, sexual behaviors of risk, greater attraction to sexist couples and to idealized love and the love–abuse bond, greater emotional dependence on the partner, and poorer quality relationships [28].

The results of our study can contribute to clarifying the unknowns that still exist about factors related to problems of gender violence and inequality in adolescents, and to the development of effective prevention programs in schools and the health field.

## 5. Conclusions

Adolescents have explicit and implicit sexist attitudes. Such attitudes exist to a greater extent in males, those born outside Spain, those who have had greater exposure to pornographic content, those who have studied in public schools, and those whose parents did not undergo university studies.

The sociological and psychological processes through which these variables contribute to the development of sexist attitudes are unclear. Parallel to the promotion of a broader research agenda on the subject, it seems advisable to reinforce education on equality and the prevention of gender violence in adolescents, especially in males, and to develop affective-sexual education programs to prevent the harmful consequences of pornography consumption in this age group.

## Figures and Tables

**Table 1:** Sociodemographic profile of participants.

Variable	Variable Values	N	%
Sex	Women	368	51.0
	Men	353	49.0
	Missing values	2	
Home country	Spain	574	80.4
	Other countries	140	19.6
	Missing values	9	
Education center	Public	582	80.5
	Private	141	19.5
Course	First course Bachillerato	601	83.1
	Second course Bachillerato	122	16.9
Type of discipline	Science	324	45.1
	Humanities	394	54.9
	Missing values	5	
Mother’s education	Non-university	491	68.7
	University	224	31.3
	Missing values	8	
Mother’s employment status	Active	605	84.6
	Non active	110	15.4
	Missing values	8	
Mother lives with a partner	No	160	22.4
	Yes	555	77.6
	Missing values	8	
Father’s education	Non-university	517	74.8
	University	174	25.2
	Missing values	32	
Father’s working status	Active	611	88.7
	Non active	78	11.3
	Missing values	34	
Father lives with a partner	No	120	17.4
	Yes	569	82.6
	Missing values	34	

**Table 2:** Factorial analysis. Bold components correspond to the original variables with higher weight in each factor.

Component	Inherent Values	% of the Variance	% Cumulated	Variables	Rotated Component Matrix		
1	2856	35.695	35.695		1	2	3
2	1574	19.679	55.374	Video gender violence	**0.882**	−0.022	−0.015
3	1135	14.190	69.564	Video sexual abuse	**0.770**	−0.154	−0.018
				Story gender violence	**0.799**	−0.104	0.048
				Story sexual abuse	**0.600**	−0.391	0.168
				Video Boy’s conduct	−0.289	**0.750**	0.163
				Story Boy’s conduct	−0.042	**0.912**	−0.010
				Video Girl’s conduct	0.063	0.155	**0.821**
				Story Girl’s conduct	0.009	−0.057	**0.875**

**Table 3:** Score of variables that measure sexist attitudes, according to the sociodemographic variables of the sample.

	Explicit Attitudes				Implicit Attitudes			
	EVAMVE		EARG		Factor 1		Factor 2	
**Variables**	m ± sd	*p*	m ± sd	*p*	m ± sd	*p*	m ± sd	*p*
Sex:								
Male (*n* = 330) Female (*n* = 353)	31.1 ± 8.6 25.5 ± 5.0	0.000	33.2 ± 8.6 26.9 ± 6.2	0.000	16.8 ± 7.2 13.4 ± 6.9	0.000	3.3 ± 2.4 2.6 ± 2.1	0.000
Country of birth:								
Spain (*n* = 547) Others (*n* = 135)	27.4 ± 6.7 31.5 ± 9.3	0.000	29.2 ± 7.5 32.7 ± 9.6	0.000				
Type of school:								
Public (*n* = 552) Private (*n* = 131)			30.3 ± 8.4 28.5 ± 6.8	0.010	15.3 ± 7.1 13.9 ± 7.5	0.036		
Mother’s education:								
Non-university (*n* = 468) University (*n* = 215)					15.4 ± 7.4 14.1 ± 6.7	0.025		
Father’s education:								
Non-university (*n* = 495) University (*n* = 168)					15.4 ± 7.4 13.8 ± 6.2	0.006		

EVAMVE: Identification of sexist attitudes, violence, and gender stereotypes (20 would indicate that they do not have any sexist attitudes and 100 that they have all the sexist attitudes measured by the scale). EARG: Identification of gender role attitudes (20 would indicate that they do not have any sexist attitudes and 100 that they have all the sexist attitudes measured by the scale). Factor 1: Identification of gender violence and sexual abuse in the video and the story (4 would indicate that they do not have a sexist attitude and 40 that they have a totally sexist attitude). Factor 2: Evaluates the behavior of the boy in the video and story (2 would indicate that they do not have a sexist attitude and 20 that they have a totally sexist attitude).

**Table 4:** Items regarding pornographic consumption.

Items
Have you ever consumed pornography?
Have you consumed pornography during the last 30 days?
Do you consider that sexual practices seen in pornography are similar to normal sexual practices?
Do you believe that pornography has had an influence on your relationship with your partner?

**Table 5:** Distribution of the sample in the levels of the independent variable “Exposure to pornography”.

Level	N	%
0.00 (very low)	50	10.9
0.25 (low)	167	36.3
0.50 (medium)	153	33.3
0.75 (high)	60	13.0
1.00 (very high)	30	6.5

## Data Availability

Data were generated during the study.

## Appendix A

Self-made story: Beatriz, 16, goes out one day to party with her friends and her best friend introduces her to a 19-year-old boy, Pedro. Pedro invites her to go out another day and she happily agrees. That day they go for a drink and Pedro accompanies her home. Taking advantage of the fact that Beatriz’s parents are away, Beatriz invites him to go up to her house to watch a movie. Although Pedro initially doesn’t want to, because he’s short of time, he finally agrees. When they get home, Pedro kisses her and they both begin to undress. They both go to Beatriz’s room and start having sex. Shortly after starting, Beatriz tells him to stop, she tells him several times, she even tells him that he is hurting her and asks him to stop. Pedro doesn’t listen to her and continues, in the meanwhile Beatriz cries, until he feels satisfied. At the end, he strokes her hair and tells her that he is in a hurry and has to go. Beatrice is left crying.
